# Supplementary material for: Localized interlayer excitons in MoSe2–WSe2 heterostructures without a moiré potential
Source: Nat Commun. 2022 Sep 12;13:5354. doi: 10.1038/s41467-022-33082-6 (PMC9468147; doi:10.1038/s41467-022-33082-6)
Supplement: Supplementary file 1 — Supplementary Information [file 41467_2022_33082_MOESM1_ESM.pdf]

Supplementary Information for

Localized Interlayer Excitons in $MoSe_2$-WSe_2 Heterostructures without a Moiré Potential

Author Names: Fateme Mahdikhanysarvejahany[1], Daniel N. Shanks[1], Matthew Klein[1], Qian Wang[2] , Michael R. Koehler[3], David G. Mandrus[4-6], Takashi Taniguchi[7], Kenji Watanabe[8], Oliver L.A. Monti[1,9], Brian J. LeRoy[1], and John R. Schaibley[1]

Author Addresses:

[1]Department of Physics, University of Arizona, Tucson, Arizona 85721, USA

[2]Guangdong Provincial Key Laboratory of Quantum Metrology and Sensing & School of Physics and Astronomy, Sun Yat-Sen University (Zhuhai Campus), Zhuhai 519082, China

[3]IAMM Diffraction Facility, Institute for Advanced Materials and Manufacturing, University of Tennessee, Knoxville, TN 37920

[4]Department of Materials Science and Engineering, University of Tennessee, Knoxville, Tennessee 37996, USA

[5]Materials Science and Technology Division, Oak Ridge National Laboratory, Oak Ridge, Tennessee 37831, USA

[6]Department of Physics and Astronomy, University of Tennessee, Knoxville, Tennessee 37996, USA

[7]International Center for Materials Nanoarchitectonics, National Institute for Materials Science, 1-1 Namiki, Tsukuba 305-0044, Japan

[8]Research Center for Functional Materials, National Institute for Materials Science, 1-1 Namiki, Tsukuba 305-0044, Japan

[9]Department of Chemistry and Biochemistry, University of Arizona, Tucson, Arizona 85721, USA

Corresponding Author: John Schaibley, johnschaibley@email.arizona.edu

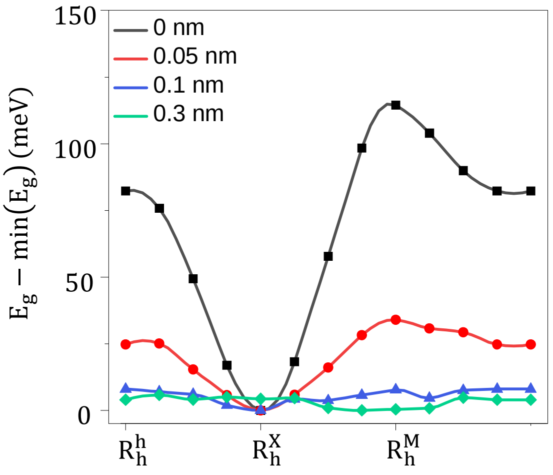

Supplementary Fig. 1: Interlayer band gap for $MoSe_2$-WSe_2 heterostructure.

Density functional theory simulation of the R-type stacking $MoSe_2$-WSe_2 heterostructure shows the moiré potential's change with increasing the vertical distance between layers. E_g is the interlayer band gap, the x-axis labels the high symmetry points of the moiré. The moiré potential disappears when the interlayer distance is increased more than 0.3 nm from the equilibrium separation.

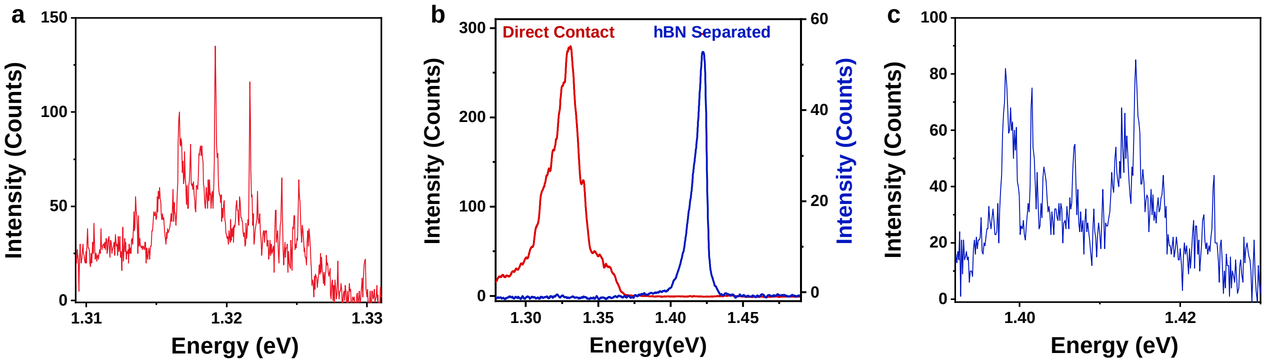

Supplementary Fig. 2: IX photoluminescence in separate regions using confocal pinhole. **a,** PL emission of the hBN separated region using 1.74 eV laser with 10 nW power and a confocal pinhole (2 μm resolution) shows an inhomogeneous distribution of narrow lines consistent with the measurement reported without pinhole. **b,** PL spectrum taken from the middle of the direct contact region (red) and hBN separated area (blue) using confocal spectroscopy with higher power showing the high energy signal originates in the hBN separated region while the low energy signal originates from the DC area. The data is taken with 50 μW laser power. **c,** Low power PL spectrum of the DC area with the pinhole (100 nW, 1.74 eV laser excitation).

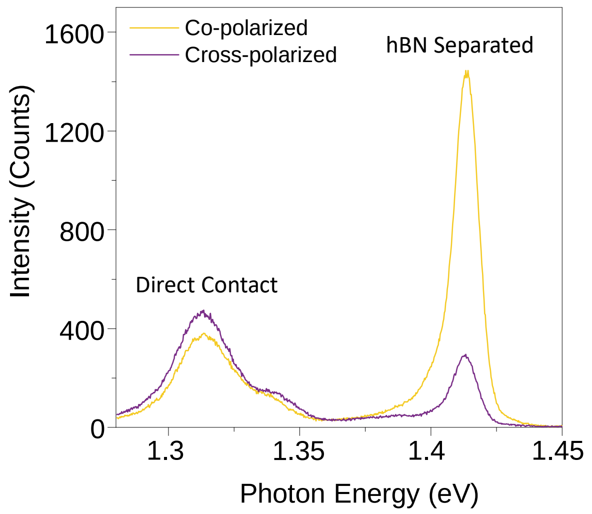

Supplementary Fig. 3: Direct contact and hBN separated polarization on another sample. Circularly polarized PL on another R-type sample. A 1.72 eV laser excited the hBN separated area while PL was collected from both regions. The PL from the hBN separated region centered at 1.41 eV shows co-circularly polarized dominant emission while the DC region's PL at 1.31 eV is cross-circularly polarized. The higher excitation power (20 µW) for this measurement results in the disappearance of the narrow lines and emergence of the wide PL spectrum.

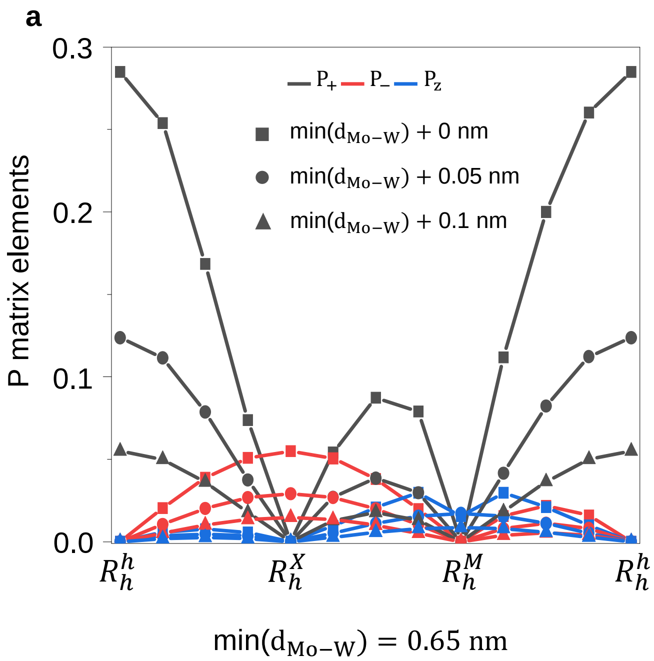

Supplementary Fig. 4: Calculated oscillator strength as a function of separation in $MoSe_2$-WSe_2 heterostructure.

Calculation of the oscillator strength for different symmetries and under various interlayer separations. At the three high-symmetry locations R_h^h, R_h^X, and R_h^M, the polarizations are dominated by P+, P- and Pz, resulting in co-, cross- and z-polarized PL.

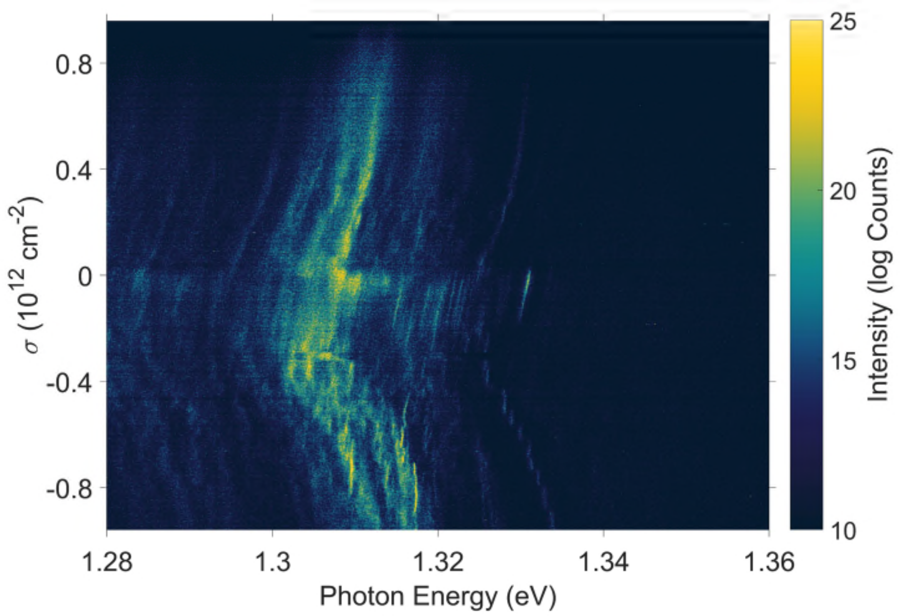

Supplementary Fig. 5: Fine structure in direct contact region.

High resolution charge density dependent PL map of the DC region shows fine structure that is attributed to the Coulomb staircase[13]. The high energy line at 1.335 eV is shows ~10 energy steps by increasing the doping of the system from -0.2×10^{12} to -0.9×10^{12} cm^{-2}.

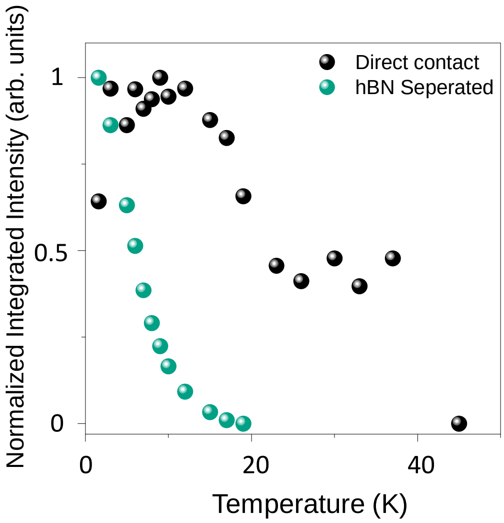

Supplementary Fig. 6: Temperature dependent PL.

Higher temperature PL measurement shows that PL in DC region persists to ~40 K.
